# Enhancing accuracy and interpretability of multi-steps water demand prediction through prior knowledge integration in neural network architecture

**DOI:** 10.1016/j.wroa.2024.100247

**Published:** 2024-08-10

**Authors:** Zhengheng Pu, Deke Han, Hexiang Yan, Tao Tao, Kunlun Xin

**Affiliations:** a College of Environmental Science and Engineering, Tongji University, Shanghai, 200092, China; b Smart Water Joint Innovation R&D Center, Tongji University, Shanghai, 200092, China; c Qingdao Haide Engineering Group, Qingdao, 260043, China

**Keywords:** Short-term water demand forecasting, Long-short term memory neural network, Convolutional Neural Network, Multi-steps forecasting, Data-driven models, Water supply system managements

## Abstract

In the field of water supply management, multi-steps water demand forecasting plays a crucial role. While there have been many studies related to multi-steps water demand forecasting based on deep learning, little attention has been paid to the interpretability of forecasting models. Aiming to improve both the forecasting accuracy and interpretability of the model, a novel urban water demand forecasting neural network (UWDFNet) was presented in this paper. Compared with traditional deep learning models, it innovatively considered domain-specific prior knowledge from water supply management and incorporated the correlation relationship between different input variables into the design of the neural network structure, and verified the consistency between the knowledge learned by the model and prior knowledge through interpretability analysis. Additionally, a systematic performance evaluation was conducted and proved that UWDFNet possesses better accuracy and stability compared to other baseline models(e.g., gated recurrent unit network (GRUN), GRUN with a corrected Network (GRUN+CORRNet), GRUN+PID, GRUN+Kmeans).

## Highlights

• Use a new model to improve the accuracy and interpretability of longterm urban water demand forecasting.
• Model interpretability is assessed with LIME to confirm the correct mapping between inputs and outputs.
• Real water demand data from a Shanghai region demonstrates that the UWDFNet achieving state-of-the-art performance.

## Graphical abstract

**Figure:**
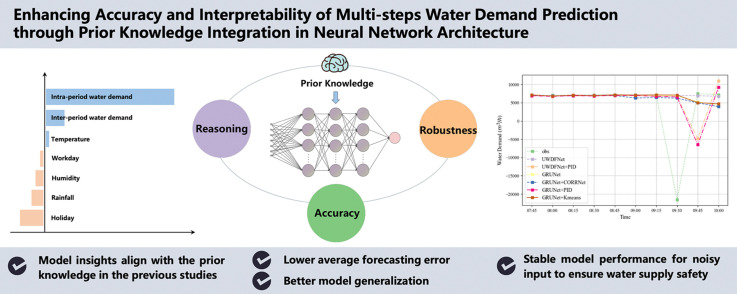

## 1 Introduction

With the development of computer science and artificial intelligence technologies, the digitalization and intelligence of water supply system management is an inevitable trend for the future. Among these, urban water demand forecasting, as a classical topic in water supply system research, is crucial to numerous water supply application scenarios driven by algorithms, such as pump scheduling optimization (Jung, Kang, Kang, Kim, 2015, Kang, Kim, Lee, Lee, Kwak, Im, 2015, Kühnert, Gonuguntla, Krieg, Nowak, Thomas, 2021, Luna, Ribau, Figueiredo, Alves, 2019), anomaly detection (Candelieri, 2017, González-Vidal, Cuenca-Jara, Skarmeta, 2019, Jian, Gao, Xu, 2022), water supply system planning (Ghiassi, Zimbra, Saidane, 2008, Rinaudo, 2015), and so on.

In the early stages of water demand forecasting research, forecasting algorithms were less of a priority than the features that drive water demand variation. Extensive research has been conducted by numerous researchers on the determinants of water demand variation, and the impacts of different exogenous variables on water demand variations have been quantified using techniques such as correlation analysis and linear regression (Adamowski, 2008, Gato, Jayasuriya, Roberts, 2007, Haque, Rahman, Goonetilleke, Hagare, Kibria, 2015, Praskievicz, Chang, 2009).

In terms of forecasting models, multiple linear regression (MLR) and autoregressive integrated moving average (ARIMA) models are the most prevalent techniques (Bakker, Vreeburg, van Schagen, Rietveld, 2013, Caiado, Caiado, 2010, Gato, Jayasuriya, Hadgraft, Roberts, 2005). These models have simple structures and excellent interpretability, but they struggle to precisely account for nonlinear variations in the water demand. In order to achieve more accurate forecasts, more complex nonlinear models, such as support vector machines (SVMs) (Candelieri, Giordani, Archetti, Barkalov, Meyerov, Polovinkin, Sysoyev, Zolotykh, 2019, Ji, Wang, Ge, Liu, 2014, Vijai, Sivakumar, 2018), random forests (RFs) (Chen, Long, Xiong, Bai, 2017, Smolak, Kasieczka, Fialkiewicz, Rohm, Siła-Nowicka, Kopańczyk, 2020, Vijai, Sivakumar, 2018), artificial neural networks (ANNs) (Ghiassi, Zimbra, Saidane, 2008, Jain, Kumar Varshney, Chandra Joshi, 2001, Jain, Ormsbee, 2002), and other machine learning models (Mouatadid, Adamowski, 2017, Oliveira, Boccelli, 2017, Xu, Zhang, Long, Chen, 2018), have been applied to water demand forecasting. By comparing the performance of the different models, numerous studies have demonstrated the superiority of these machine learning algorithms (Antunes et al., 2018). It is also worth mentioning that at this stage, as a result of the increased capability of machine learning models to model nonlinear systems, the prediction frequency has shifted from the early daily level to the hourly level, which means that the water utilities can make hourly decisions in real-time based on the forecasts. Later on, together with the technological innovation of deep learning algorithms in many domains, various deep learning algorithms for sequence modeling (e.g., GRU, LSTM) have been widely implemented for short-term water demand forecasting (Chen, Yan, Yan, Wang, Tao, Xin, Li, Pu, Qiu, 2022, Du, Zhou, Guo, Guo, Wang, 2021, Guo, Liu, Wu, Li, Zhou, Zhu, 2018, Kühnert, Gonuguntla, Krieg, Nowak, Thomas, 2021, Salloom, Kaynak, He, 2021). Deep learning algorithms are generally considered to have stronger nonlinear modelling and feature extraction capabilities and consequently have better performance compared to linear models and machine learning models. In addition, many hybrid models combining deep learning with enhancement techniques (e.g., wavelet decomposition (Du, Zhou, Guo, Guo, Wang, 2021, Pu, Yan, Chen, Li, Tian, Tao, Xin, 2023), cluster algorithm (Salloom et al., 2021), and PID control (Salloom et al., 2022)) have been proposed to further increase forecasting accuracy.

Clearly, technological advances in forecasting models have led to an ongoing increase in the accuracy of water demand forecasts. However, in practical applications, multi-step short-term water demand forecasting still remains a challenge. On the one hand, current research mostly focuses on single-step water demand forecasting, while multi-steps forecasting is often performed iteratively based on the single-step forecast. As a result, the model’s accuracy fails to meet practical requirements due to error accumulation. On the other hand, the current research pays limited attention to the interpretability of deep learning models, which leads to a lack of interpretation and understanding of model results by decision makers and raises some potential uncertainty risks.

In this paper, we aim to enhance the precision and interpretability of the multi-steps water demand forecasting model. To achieve this, a novel urban water demand forecasting neural network (UWDFNet) was developed. It combines two innovations: (1) A tailored RNN-based architecture to capture complex, long-range dependencies in the observational data and adopt a many-to-many input-output approach to avoid error accumulation from iterative prediction; and (2) an innovative modelling approach based on a gating mechanism is proposed to capture the intrinsic correlations between historical water demand, exogenous variables, and water demand by incorporating prior knowledge.

For (1) and (2), this study first developed two separate convolutional gated recurrent units (CGRU) block, specifically for extracting the temporal dependencies in the intra-period water demand and the inter-period water demand, respectively. Subsequently, a gating linear unit (GLU) is employed to adaptively control the transmission and updating of temporal information at different scales, enabling the model to flexibly capture the influences of long-term dependencies and short-term fluctuations on water demand variations. Furthermore, the exogenous variables are adaptively encoded as multiplicative correction factors for the forecasted water demand through the gating mechanism, thus representing their impact on the variations in the forecasted water demand.

Further, we compared and analyzed the accuracy, sensitivity and interpretability of the models using real water demand data in a region of Shanghai. Overall, the main contributions of this paper can be summarized as follows:

1. A novel end-to-end water demand forecasting model, UWDFNet, was proposed specifically designed for multi-steps forecasting. To the best of our knowledge, this is the first water demand forecasting model that considers the interdependencies among input variables at the level of model architecture design.
2. By employing the Local interpretable model-agnostic explanations(LIME) method, the interpretability analysis was conducted to validate that UWDFNet captures the correct correlation between the different inputs and outputs.
3. Real water demand data from a specific region in Shanghai was used to demonstrate that the UWDFNet model achieves state-of-the-art performance compared to baseline models, even after adjusting for the forecasts of the baseline models.

## 2 Results and discussion

### 2.1 Model performance analysis

To evaluate the prediction performance of the UWDFNet model, the four baseline models mentioned in Section 4.5 (gated recurrent unit network (GRUN) (Guo et al., 2018), GRUN with a corrected neural network(GRUN+CORRNet) (Guo et al., 2018), GRUN+PID (Salloom et al., 2022), GRUN+Kmeans (Salloom et al., 2021)) were compared with it. Furthermore, considering the interference of high-frequency noise and previous research demonstrating that wavelet decomposition can avoid the interference of high-frequency signals on predictions by extracting components of different frequency domains (Pu et al., 2023), this study also compared the effects of adding wavelet adaptive decomposition to short-term water demand forecasting. The performance of these models on the test dataset is summarized in Table 1.

**Table 1:** Prediction performance on the test dataset using urban water demand forecasting neural network (UWDFNet), UWDFNet without wavelet transform (UWDFNet+NW), UWDFNet+PID, gated recurrent unit network (GRUN), GRUN with a corrected neural network (GRUN+CORRNet), GRUN+PID, GRUN+Kmeans).

	UWDFNet	UWDFNet+NW^a^	UWDFNet+PID	GRUN	GRUN+CORRNet	GRUN+PID	GRUN+Kmeans
MAPE(%)	2.98	3.20	2.48	3.75	3.74	2.77	3.35
MAE	203.1047	217.9159	168.4992	254.7429	255.1814	187.3841	224.4270
Acc	0.7574	0.7398	0.8010	0.7331	0.7281	0.7792	0.7098
Corr	0.8978	0.8881	0.9289	0.8522	0.8634	0.9204	0.8793

a NW represents not considering adaptive wavelet decomposition

**Fig. 1:**
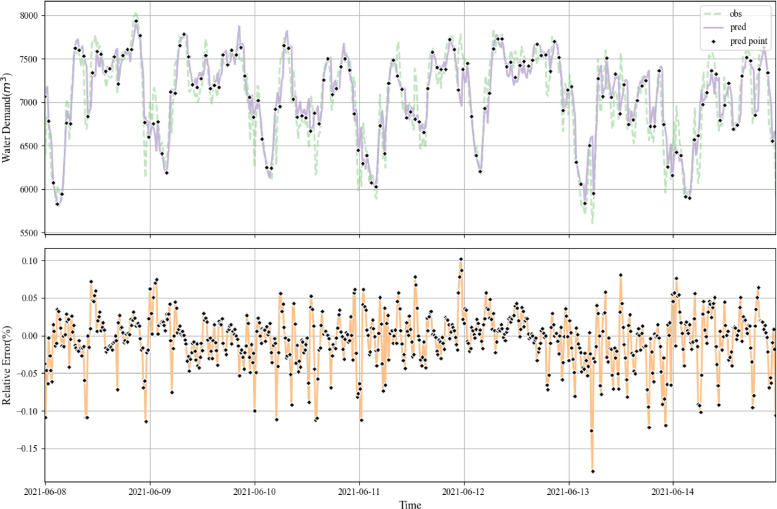
Multi-step water demand forecasting performance of the UWDFNet+PID model. The upper subplot illustrates the comparison between observed (obs) and predicted (pred) water demand over time. Each ”pred point” indicates the starting point of a new prediction sequence. The lower subplot depicts the relative error between between the predicted and observed water demand.

From the results, the following observations can be made: Firstly, compared to the baseline model and the other correction methods, the proposed UWDFNet in this study achieves better performance, while the combination of UWDFNet and PID achieves the best forecast results with the MAPE 2.48 %, shown as Fig 1. Secondly, comparing the performance of different enhancement techniques, it can be found that GRUNet+PID > GRUNet+Kmeans > GRUNet+CORRNet. The improvements of GRUNet+Kmeans and GRUNet+PID forecast accuracy(mape) are very significant, contributing 10.6 % and 26.1 %, respectively. The improvement of GRUNet+CORRNet is not significant, which might be due to the fact that the deviation pattern between the hourly water demand predictions and the actual values is more complex compared to the daily water demand. Thirdly, the results also show that separating the high-frequency noise by adding wavelet adaptive decomposition can indeed enhance the performance of the model forecasts.

**Fig. 2:**
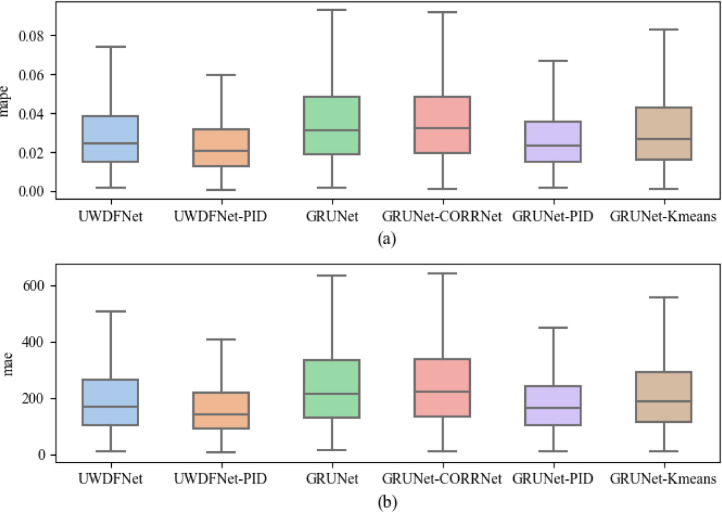
Distribution of forecast errors of different models on the test dataset.

To assess the performance of each sample on different models, the comparison of forecast error distributions on different models was conducted, as shown in Fig 2. Observations revealed that UWDFNet exhibited a narrower error distribution interval compared to GRUNet, GRUNet+CORRNet, and GRUNet+Kmeans, suggesting that UWDFNet displayed better forecast stability. Furthermore, UWDFNet+PID also demonstrated a superior error distribution compared to GRUNet+PID.

**Fig. 3:**
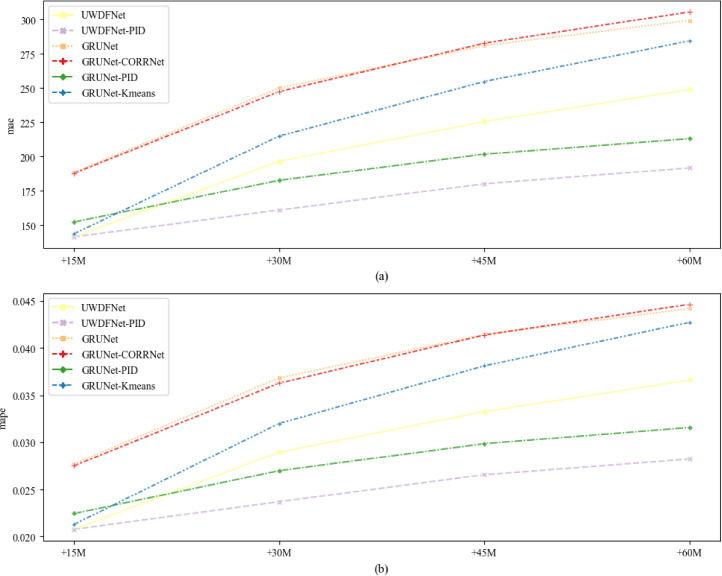
Stepwise average forecast error for multi-steps water demand forecasting with different models.

In addition, through a comparative analysis of the stepwise forecast errors among different models, a comprehensive investigation into the temporal characteristics of model prediction accuracy can be conducted, as depicted in Fig 3. The comparative results demonstrate that UWDFNet outperforms GRUNet, GRUNet+CORRNet, and GRUNet+Kmeans at each time step. This indicates that the adoption of a many-to-many structure can mitigate error accumulation and consequently yield better performance in contrast to iterative prediction. It’s worth noting that the performance of GRUNet+CORRNet and GRUNet+Kmeans weaken over time, while the performance of GRUNet+PID improves. This suggests that PID as a correction method has a more significant contribution to improving long-term forecast accuracy.

### 2.2 Sensitivity analysis

**Fig. 4:**
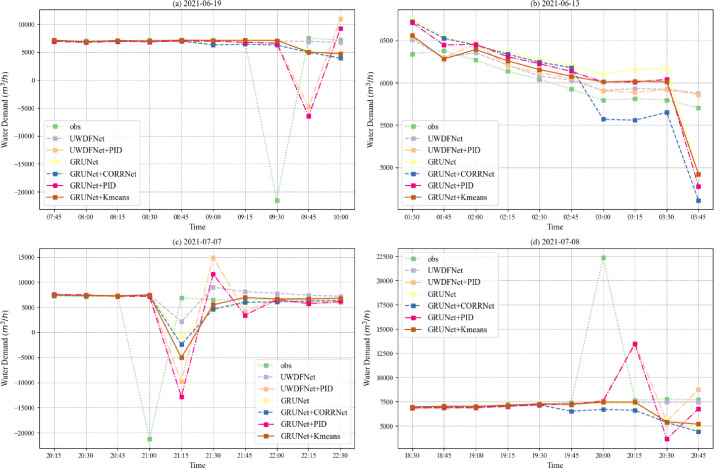
The forecasting performance of different models when presented with input containing high-frequency noise.

In practical application, it is crucial to take into account the impact of abnormal monitoring data. In order to assess the stability of the forecasting performance of the different models when presented with anomalous inputs, high-frequency noise was randomly added to the historical water demand data, and then a comparative analysis was conducted to evaluate the forecasting performance of different models with inputs containing high-frequency noise, as illustrated in Fig. 4.

Some observations can be derived from the figure: (1) Compared to other baseline models, UWDFNet demonstrates a more stable and accurate forecasting performance in scenarios involving multiple inputs with high-frequency noise, exhibiting minimal susceptibility to anomalous inputs;(2) The PID correction method is highly susceptible to the influence of high-frequency noise, leading to significant forecasting errors.

### 2.3 Model interpretability analysis

In the application of deep learning models, despite the high accuracy achieved by the model, a potential problem is whether the deep learning model learns the correct knowledge from the data due to its black-box properties. In the field of water demand forecasting, there has been a large amount of literature on the features that influence water demand (Adamowski, 2008, Hu, Tong, Wang, Yang, de Oliveira Turci, 2019, Xenochristou, Kapelan, Hutton, Hofman, 2017), including two main categories, one is the intra-period water demand and the inter-period water demand, and the other is the meteorological features, including temperature, humidity, and rainfall, in which the temperature has a positive correlation with water demand, while rainfall and humidity have a negative correlation with the water demand. On this basis, the LIME method was employed to analyze the correlation of different input features on the model prediction results and to verify that UWDFNet learns the correct knowledge by checking whether the analyzed results are consistent with the prior knowledge.

**Fig. 5:**
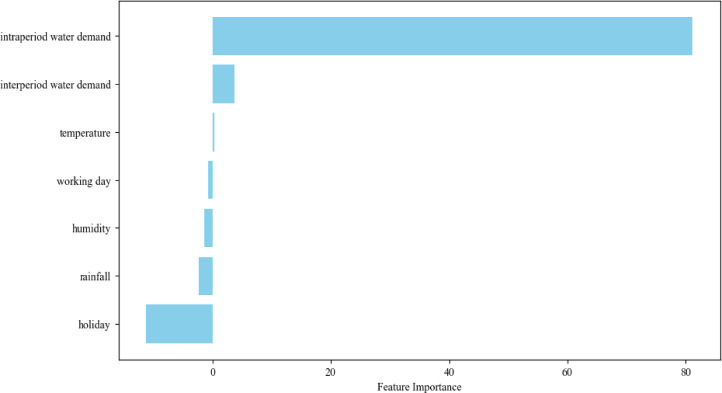
Average contributions of different input features from the test dataset to the prediction results of the model.

In this analysis, the input features of the UWDFNet model were categorized into seven categories: intra-period water demand ($Q_{t-1}$ to $Q_{t-7}$), inter-period water demand, temperature, rainfall, humidity, working days, and holidays. First, the contribution of each input feature to the prediction results was computed for each sample in the test dataset, represented by the corresponding regression coefficients. Then, the average contribution of input features across all samples was presented in Fig. 5.

The analysis results show that the model prediction is significantly influenced by intra-period water demand, inter-period water demand, as well as temperature, humidity, rainfall, and holidays, in which the prediction is positively correlated with temperature and negatively correlated with humidity and rainfall. Regarding the influence of holidays, although the regression coefficients of dummy variables may not hold absolute significance, it can be observed that holidays have a noticeable negative contribution to the prediction compared to working days. These findings are generally consistent with the priori knowledge, and thus it can be shown that UWDFNet learns the correct correlation relationship between different input variables and predictions from the data.

## 3 Conclusion

In addressing the challenge of the multi-steps water demand forecasting, a novel neural network architecture named UWDFNet was introduced in this study by rigorously exploring the interdependencies among input variables. This architecture has demonstrated significant advantages in forecast accuracy, stability, and interpretability. UWDFNet uses a specific multi-source model input mechanism for feature extraction and employs gate mechanisms to dynamically learn the influence of different input variables on model forecasts.

To comprehensively evaluate the performance of the proposed approach, a systematic assessment has been conducted from three dimensions: accuracy, stability, and interpretability. A comparative analysis was performed against four baseline models (GRUNet, GRUNet+CORRNet, GRUN+Kmeans, GRUN+PID). The conclusions drawn from this investigation are as follows:

1. Compared to the other baseline models, UWDFNet and UWDFNet+PID exhibit lower average errors and smaller variances, indicating their superiority in the multi-steps water demand forecasting scenarios.
2. UWDFNet demonstrates enhanced resilience to abnormal noise compared to the other baseline models, showcasing robust forecast stability.
3. The contribution of different input variables to UWDFNet’s rank is as follow: Intra-period water demand > Inter-period water demand > Rainfall > Humidity > Temperature. Among these, intra-period water demand, inter-period water demand, and temperature positively influence predictions, while rainfall and humidity have negative effects. Furthermore, holidays exhibit a notable negative influence on predictions compared to workdays, aligning with the prior knowledge in the previous studies.
4. Among the three distinct model correction methods, the optimization performance ranks as follows: PID > Kmeans > CORRNet. Both CORRNet and Kmeans corrective effects gradually diminish over time, whereas PID’s correction effect strengthens progressively over time. However, PID is sensitive to data variations and its corrective performance is affected when inputs contain abnormal noise.

In the future, it is important to consider the integration of model interpretability into model assessment. Such integration could be a key aspect of model evaluation by allowing for a more rational assessment of the rationality of the model’s learning mapping relationships.

## 4 Methodology

This section introduces the UWDFNet model and discusses the model’s applicability to water demand forecasting. Firstly, the definition of the multi-steps water demand forecasting problem was formalized, and improvements were made to the original formulation by considering the interdependencies among input variables. Subsequently, a detailed introduction was provided for all components of UWDFNet (CGRU, GLU) along with their relevant knowledge. Lastly, the overall structure of UWDFNet was presented, accompanied by a comprehensive description of its workflow.

### 4.1 Multi-steps water demand forecasting formulation

Given the water demand series $X=\left\{x_{1},x_{2},x_{3},\ldots ,x_{t},\ldots ,x_{n}\right\}$, $X\in R^{n}$, exogenous variables $EV=\left\{ev_{1},ev_{2},ev_{3},\ldots ,ev_{t},\ldots ,ev_{n}\right\}$, $EV\in R^{n\times m}$, the problem of short-term urban water demand forecasting can be formulated as Eq. (1):

(1) $${\hat{X}}_{t+1:t+H}=f\left(W_{x}\cdot X_{1:t}+\sum_{i=1}^{n}W_{ev}^{i}\cdot ev_{1:t}^{i}\right)+\epsilon$$

Where ${\hat{X}}_{t+1:t+H}$ is the forecast water demand series $X=\left\{x_{t+1},\ldots ,x_{t+H}\right\}$, $W_{x}$ and $W_{ev}^{i}$ represent the weight matrix developed for the problem with the variable X and exogenous variables $EV=\left\{ev^{1},ev^{2},\ldots ,ev^{n}\right\}$, respectively. f represent the non-linear function approximated by the model, H is the intended forecasting horizon and ϵ denotes the estimated residuals.

As mentioned in the Eq. (1), the main features influencing the water demand variation include historical water demand, weather, and holidays, where weather and holidays are exogenous variables, while historical water demand involves two types of temporal variations within a recent period and with the same phase among different periods, namely intra-period water demand and inter-period water demand. Therefore, the above equation can be further specified as Eq. (2):

(2) $$\begin{array}{c} {\hat{X}}_{t+1:t+H}=f(W_{x}\cdot X_{1:t}+\sum_{k}W_{x_{T_{k}}}\cdot X_{t+1-\mathrm{kT}:t+H-\mathrm{kT}}\\ \;+W_{m}\cdot M_{1:t}+W_{T}\cdot T_{1:t})+\epsilon \end{array}$$

where $W_{x_{T}}$, $W_{m}$ and $W_{T}$ represent the weight matrix developed for the problem with the intra-period water demand($X_{1:t}$), inter-period water demand ($X_{t+1-kT:t+H-kT}$), weather ($M_{1:t}$) and holidays ($T_{1:t}$), respectively. Typically, T denotes the daily period.

Nevertheless, In the process of model development, the direct integration of meteorological data as input variables presents several challenges. First, the inherent inaccuracies in spatial resolution and temporal granularity of meteorological data can introduce undesired noise into the model. Second, the additive model struggles to capture the interactive relationship between the exogenous variables and the variation of water demand. Third, introducing the exogenous variables into the model as uninformative features within the homogeneous structure of water demand data can negatively affect the model performance (Grinsztajn et al., 2022). In light of these considerations, a multiplicative model rather than an additive model was proposed as Eq. (3)-Eq. (7):

(3) $$\begin{array}{c} {\hat{X}}_{v_{t+1:t+H}}=f\left(k_{x}f_{x}\left(X_{1:t}\right)+\left(1-k_{x}\right)f_{x_{T}}\left(\sum_{k}X_{t+1-\mathrm{kT}:t+H-\mathrm{kT}}\right)\right) \end{array}$$

(4) $$\begin{array}{c} {\hat{X}}_{t+1:t+H}=k_{T}\cdot k_{m}\cdot {\hat{X}}_{v_{t+1:t+H}}+\epsilon \end{array}$$

(5) $$\begin{array}{c} k_{x}=\sigma \left(W_{x}f_{x}\left(X_{1:t}\right)+b_{x}\right) \end{array}$$

(6) $$\begin{array}{c} k_{m}=\sigma \left(W_{m}M_{1:t}+b_{m}\right) \end{array}$$

(7) $$\begin{array}{c} k_{T}=\sigma \left(W_{T}T_{1:t}+b_{T}\right) \end{array}$$

In this formulation, a virtual water demand, denoted as ${\hat{X}}_{t+1:t+H}^{v}$ is hypothesized to be solely influenced by intra-period water demand $X_{1:t}$ and inter-period water demand $X_{t+1-kT:t+H-kT}$ at each time point. The coefficient $k_{x}$ represents the impact of intra-period water demand on ${\hat{X}}_{t+1:t+H}^{v}$, which can be parameterized as Eq. (5), where σ is a logistic sigmoid function, $W_{x}$ and $b_{x}$ are the parameters to be trained. While $1-k_{x}$ represents the impact of inter-period water demand on ${\hat{X}}_{t+1:t+H}^{v}$. This is because the fact that the intra-period water demand and the inter-period water demand have different impacts on the virtual water demand under different conditions of water demand variations. For instance, intra-period water demand predominantly influences the virtual water demand in trending water demand variation while inter-period water demand play a dominant role in periodic water demand variation. Non-linear mapping functions, $f_{x}$ and $f_{x_{T}}$, are utilized to encode the intra-period and inter-period water demand, respectively. Furthermore, the virtual water demand is multiplied by the weather modification coefficient $k_{m}$ and the holiday modification coefficient $k_{T}$ to reflect the influence of weather and holidays on the water demand variations, which also can be parameterized as Eq. (6) and Eq. (7), and ultimately yielding the forecast.

### 4.2 GLU layer

Gating mechanism is important concepts widely used in recurrent neural networks (RNNs), which control the path through which information flows in the neural network, enabling effective processing of sequential data. Long Short-Term Memory (LSTM) networks, for instance, incorporate mechanisms such as input and forget gates to model long-term memory (Hochreiter and Schmidhuber, 1997). Gated Linear Unit (GLU) are simplified gating mechanism that have found extensive applications in natural language processing (NLP) (Dauphin, Fan, Auli, Grangier, 2017, Kalchbrenner, Espeholt, Simonyan, Oord, Graves, Kavukcuoglu, 2016).

The main idea of GLU is to couple linear units with gating mechanism, introducing a form of nondeterministic gating. Specifically, the expression for GLU is given by Eq. (8):

(8) $$h_{l}\left(X\right)=\left(X*W+b\right)⊗\sigma \left(X*V+c\right)$$

Where X represents the input, W and V are the learnable weight matrices, b and c are the bias terms. The design of GLU has two main advantages. First, it facilitates the flow of gradients between network layers by an undecayed gradient path, which can be seen as a form of multiplicative skip connection. This enables the gradient to propagate effectively through the network layers, addressing the vanishing gradient in neural networks.

Second, GLU separates information transmission from information encoding. Information transmission is controlled by the gating unit, while information encoding is handled by the linear unit. This design allows the model to flexibly control when to activate information transmission and how much information was transmitted, which helps the model better fit the smoothing function with latent variables.

### 4.3 UWDFNet

**Fig. 6:**
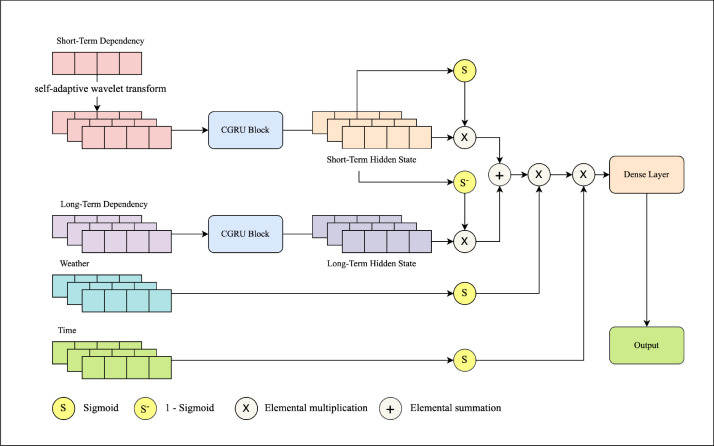
Schematic of the UWDFNet architecture for water demand forecasting.

The main idea of UWDFNet is to employ CGRU blocks in conjunction with a gating mechanism to model the mapping of historical water demand, exogenous variables and future water demand in Fig 6. The CGRU is made to capture the temporal dependencies that are present in historical water demand data. The gating mechanism, which uses real-world physical logic, encodes other outside variables into soft gates to show how these variables affect water demand variation. This approach effectively integrates prior knowledge pertinent to water demand forecasting tasks into the network structure design, and Fig 6 illustrates the UWDFNet architecture proposed in this paper.

In this process, intra-period water demand, inter-period water demand, weather, and holidays are fed into the network as inputs. The CGRU Block consists of a one-dimensional convolutional layer (Conv1D) and a gated neural unit network layer (GRU), corresponding to $f_{x}$ and $f_{x_{T}}$ in the Eq. (3), serves as an encoder to capture the temporal dependencies in the intra-period and inter-period water demand. The GLU layer implicitly controls the impact of intra-period water demand and inter-period water demand on the variations in future water demand by encoding the latent temporal features of the intra-period water demand as a soft gate to represent $k_{x}$. As for the exogenous variables (weather and holiday), they are encoded into soft gates to represent weather correction coefficients $k_{T}$ and holiday correction coefficients $k_{m}$ through a gating mechanism, which simulates the impact of weather and holidays on the variations in future water demand. It is noted that the holiday is a categorical variable (representing whether the current time is a working day), therefore, it needs to be embedded into continuous numerical vectors to ensure the effectiveness of gradient updates in the neural network. Finally, the output result is obtained through a linear transformation in the dense layer.

### 4.4 PID correction

The PID method is used in industrial control systems to correct the system’s output, making it follow the expected target or trajectory. Its specific formula can be written in the following form:

(9) $$u_{t}=-K_{p}e_{t}-K_{i}\sum_{x=0}^{x=t}e_{x}-K_{d}\left(\Delta e\right)$$

Where $e_{t}$ represents the system error of the output at step t, $K_{p}$ is a proportionality gain, $K_{i}$ is a positive number representing the integral gain, and $K_{d}$ represents the derivative gain. The system’s final output $P_{t}$ becomes as follows:

(10) $$\begin{array}{cc} & P_{t}=PV_{t}+u_{t}\\ & e_{t}=P_{t}-RV_{t} \end{array}$$

When forecasting multi-steps water demand, the process of using PID for forecast correction is shown in Eq. (11). First, the prediction $PV_{t}$ is generated through the neural network. Then, the PID method is used to correct the error in conjunction with the previous error information. The corrected forecast result will be used for calculating the error information with the observations to correct subsequent forecasts. Another issue to note is that future error information cannot be obtained when making multi-step forecasts. Therefore, some improvements have been made to the PID correction method.

(11) $$\begin{array}{cc} t\geq H: & \;u_{t}=-K_{p}e_{\left(t-T\right)}-K_{i}\sum_{\begin{array}{c} x=(i-1)T \end{array}}^{x=t-T}e_{x}-K_{d}\left(e_{\left(t-T\right)}-e_{\left(t-T-1\right)}\right)\\ t<H: & \;u_{t}=-K_{p}e_{t-1}-K_{i}\sum_{x=0}e_{x}-K_{d}\left(e_{t-1}-e_{t-2}\right) \end{array}$$

At the t-th step, if t is less than the forecast horizon T, previous error information can be used. If t is greater than the T, the error information from the same step at the last forecast horizon is used.

### 4.5 Benchmark models

In addressing the water demand forecasting problem, a variety of deep learning models have been proposed. The GRUN model has demonstrated notable performance in short-term water demand forecasting tasks (Guo et al., 2018). Moreover, for multi-steps water demand forecasting problems, various enhancement techniques, including the addition of corrective networks, PID correction (Salloom et al., 2022), and K-means feature expansion (Salloom et al., 2021), have been considered effective in reducing error accumulation and further enhancing forecast accuracy. In this study, these correction methods are combined with the GRUN model to serve as benchmark models for multi-step water demand forecasting tasks, with a detailed introduction to these models provided in the Appendix A. By comparing the performance of the UWDFNet model and the benchmark models, the superiority and potential application value of the UWDFNet model in forecasting tasks can be validated.

### 4.6 Evaluation metrics

In order to systematically evaluate and compare different models, we have adopted error metrics such as Mean Absolute Percentage Error (MAPE) and Mean Absolute Error (MAE), which are widely applied in water demand forecasting. In addition, for multi-step water demand forecasting, we also employ additional metrics, namely Accuracy and Pearson correlation coefficient, to evaluate the direction as well as the correlation of water demand variation across the entire forecasting horizon, and the specific formulas for these metrics are shown as follows:

(12) $$\text{MAPE}=\frac{100\%}{n}\sum_{i=1}^{n}\left|\frac{Y_{i}-{\hat{Y}}_{i}}{Y_{i}}\right|$$

(13) $$\text{MAE}=\frac{1}{n}\sum_{i=1}^{n}\left|Y_{i}-\hat{Y}\right|$$

(14) $$\text{Acc}=\frac{1}{n}\sum_{i=1}\frac{I_{\left(Y_{i}^{t+H}-Y_{i}^{t+1}\right)*\left({\hat{Y}}_{i}^{t+H}-{\hat{Y}}_{i}^{t+1}\right)>0}}{I_{\left(Y_{i}^{t+H}-Y_{i}^{t+1}\right)*\left(Y_{i}^{t+H}-Y_{i}^{t+1}\right)>0}}$$

(15) $$\text{Corr}=\frac{\sum_{i=1}^{n}\left(Y_{i}-\bar{Y}\right)\left({\hat{Y}}_{i}-\bar{\hat{Y}}\right)}{\sqrt{\sum_{i=1}^{n}{\left(Y_{i}-\bar{Y}\right)}^{2}\sum_{i=1}^{n}{\left(Y_{i}-\bar{Y}\right)}^{2}}}$$

where $Y_{i}$ and ${\hat{Y}}_{i}$ denotes the ith observation and prediction, respectively. n denotes the number of samples. t represents the current time step, and H represents the forecast horizon. I stands for the indicator function shown as Eq. (16).

(16) $$I_{A}\left(x\right)=\left\{ \begin{array}{cc} 1 & \;\text{if}\;x\in A\\ 0 & \;\text{if}\;x\notin A \end{array}\right.$$

### 4.7 Explainability analysis

In the application of modern machine learning, particularly when using complex black-box models (such as deep learning models), the explainability of models has become an important issue. These black-box models have demonstrated superior performance in various tasks, however, their decision-making process is often difficult to comprehend. For the water supply system, ensuring the safety of the water supply is of utmost importance, thus requiring a clear understanding of the model’s decision-making process. Providing a method to interpret these complex model predictions has become a vital research direction.

LIME is a widely used algorithm in the field of machine learning, designed to offer explainability and enhance the understanding of model forecasts (Ribeiro et al., 2016). Its principle involves sampling the local space of the input, and then training a straightforward model (such as weighted linear regression) to approximate the prediction behavior of the black-box model within this local space. The parameters of this simple model (such as the weights of the linear model) can be interpreted as the contribution of each feature to the prediction, thereby helping people understand the decision rules of the black-box model in this local space.

The specific formula of LIME is as follows:

(17) $$\xi \left(x\right)=\mathrm{argmin}\lambda L\left(f,\lambda ,\pi_{x}\right)+\Omega \left(\lambda \right)$$

In this formula, ξ(x) represents the ideal local interpretive model, f denotes the black-box model, λ is the parameter of the local interpretive model, $\pi_{x}$ is a distance function measuring the distance from the sample to x, L is the loss function, which is used to measure the prediction discrepancy between f and λ under $\pi_{x}$, and Ω is the complexity measure employed to limit the complexity of λ. In practical applications, a linear model is generally chosen as λ, square loss as L, an exponential decay function as $\pi_{x}$, and the number of non-zero weights of λ as Ω.

## 5 Case study

### 5.1 Data description

Water demand data is collected from a real districted metering area(DMA) in Shanghai, China, with a daily water production of about 200,000 tons, including a water plant and two pump stations. Hourly meteorological data (including average temperature, rainfall, and relative humidity) from Shanghai are also collected as exogenous variables. Table 2 shows the statistical results of these data.

**Table 2:** Statistics of water demand dataset.

	Water Demand(${\text{m}}^{3}/\text{h}$)	Rainfall(mm)	Temperature(${}^{\circ }\text{C}$)	Humidity(%)
Mean	6369.24	0.22	19.46	74.66
Std	959.86	0.75	8.38	18.92
Min	2483.70	0.00	-8.29	15.00
25 %	5861.82	0.00	12.83	62.00
50 %	6461.39	0.00	21.65	77.50
75 %	7050.28	0.05	26.35	90.00
Max	8966.09	12.24	34.56	100.00

The dataset covers a time range from July 2020 to July 2021, comprises a total of 46749 data samples. The time resolution of water demand data is 15 minutes, while meteorological data has a time resolution of 1 hour. To maintain consistency, all data in the study are processed using linear interpolation into 15-minute intervals. The entire dataset is divided into training, validation, and test sets. The first 80 % of the data is used for training, and the remaining 20 % is divided into validation and test sets, each accounting for 10 %.

### 5.2 Model development

The UWDFNet model is proposed for multi-steps prediction of water demand. The input of the UWDFNet model consists of four parts:

1. **Intra-period water demand**: This feature contains water demand data from the past three hours, i.e., from t−1 to t−12. This feature is mainly used to capture the changes in water demand in the short term.
2. **Inter-period water demand**: This feature contains water demand data for the same forecasting horizon in the past seven days, i.e., t+1−96n,⋯,t+12−96n,n=1⋯7. This feature is mainly used to capture the cyclical variation in water demand at the current time.
3. **Weather**: This feature contains temperature, rainfall, and relative humidity data from the past three hours, i.e., from t−1 to t−12. This feature is mainly used to reflect the current weather conditions, as weather conditions may affect water demand.
4. **Holidays**: This is a categorical variable indicating whether the day is a working day. This feature helps capture the difference in water demand between working days and holidays.

During the training of the neural network, the Adam optimizer was employed, which is an adaptive learning rate optimization algorithm that combines the advantages of Momentum Gradient Descent and Root Mean Square Propagation(RMSProp) methods, to guide the updating of neural network weights (Kingma and Ba, 2014). Compared to the traditional Stochastic Gradient Descent (SGD) method, Adam converges faster and automatically adjusts the learning rate during training. This makes it more efficient and stable in practice, especially when dealing with large-scale and complex datasets. The Mean Squared Error (MSE) was used as loss function in this study, the specific formula is shown as Eq. (18):

(18) $$\mathrm{MSE}=\frac{1}{n}\sum_{i=1}^{n}{\left(y_{i}-{\hat{y}}_{i}\right)}^{2}$$

where $y_{i}$ is the actual value, ${\hat{y}}_{i}$ is the predicted value, and n is the number of data points. Moreover, early stopping strategy was implemented during train process, which monitors the loss on the validation dataset to determine whether the model should continue training. When the validation loss no longer significantly decreases within a certain number of epochs, the training will be terminated early. This helps to avoid overfitting the model on the training dataset, thereby improving the model’s generalization ability on unseen data.

As for the selection of neural network hyperparameters, in this study, the tree-structured parzen estimator (TPE) method was employed to evaluate the performance of different parameter combinations on the validation set to determine the optimal hyperparameter combination. TPE is a hyperparameter optimization algorithm proposed by James Bergstra and Yoshua Bengio in 2011 (Bergstra et al., 2011). It aims to approximate the optimal solution by establishing a probability model of the objective function and continuously updating the parameter distribution. Compared to traditional grid search method, the TPE algorithm is more efficient and capable of finding better solutions with fewer iterations. The hyper-parameter search ranges and optimization configuration of UWDFNet, GRUN and CORRNet are detailed in Table 3.

**Table 3:** The hyper-parameter search range and optimal configuration of models.

	Hyper Parameters	Search Range	Optimal configuration
	learning rate	[0.01, 0.001, 1e-4, 1e-5]	1e-4
	batchsize	[32, 64, 128, 256]	128
UWDFNet	Number of out channels	[16, 32, 64, 128, 256]	16
	Number of Nodes(GRU)	[32, 64, 128, 256]	32
	Embedding dim	[2, 4, 8, 16]	4
	Kernel size	[2, 3, 4]	3
GRUN	Number of Nodes (GRU)	[32, 64, 128, 256]	32
	Number of Nodes (Linear)	[32, 64, 128, 256]	64
	Number of Layers (Linear)	[1, 2, 3]	2
CORRNet	Number of layers	[1, 2, 3]	2
	Number of Nodes (Linear)	[4,8,16]	16
Kmeans	Number of clusters	[3, 4, 5]	3
PID^a^	Kp	-	0.4
	Ki	-	0.01
	Kd	-	0.001

a The selection of PID parameters is based on previous study (Salloom et al., 2022).

## CRediT authorship contribution statement

**Zhengheng Pu:** Writing – original draft, Visualization, Validation, Methodology. **Deke Han:** Data curation, Resources. **Hexiang Yan:** Writing – review & editing, Supervision, Methodology. **Tao Tao:** Writing – review & editing, Supervision, Methodology. **Kunlun Xin:** Writing – review & editing, Supervision, Resources, Funding acquisition, Conceptualization.

## Declaration of competing interest

The authors declare that they have no known competing financial interests or personal relationships that could have appeared to influence the work reported in this paper.

## Data Availability

The authors do not have permission to share data.

## Appendix A Appendix

### A1 One-dimensional convolution

Compared to traditional 2D convolution (Sainath et al., 2015), Conv1D layer means the convolution kernel that is convolved with the layer input over a single spatial or temporal dimension to produce output. The expressions of the one-dimensional convolution operation are shown in Eq. (A.1)

(A.1) $$\begin{array}{c} y_{p}^{\left(l+1\right)}=f\left(y_{p}^{\left(l\right)}⊗\mathrm{rot}180\left(\omega_{p}^{\left(l\right)}\right)+b_{p}^{\left(l\right)}\right) \end{array}$$

where $y^{\left(l\right)}$ denotes the activation value of the 1 -th layer; $w_{p}^{\left(l\right)}$ and $b_{p}^{\left(l\right)}$ are the weight and bias matrices, respectively; ⊗ is the symbol of convolutional operation; rot180(ø) means to rotate the input by $180^{\circ }$.

### A2 Gated recurrent unit

gated recurrent unit(GRU) network is a variant of RNN (Chung et al., 2014), which introduces a reset gate and an update gate into the network structure to reduce the gradient vanishing problem. Specifically, as shown in Fig., the reset gate $r_{t}$ regulates how much information about the hidden state of the last moment $h_{t-1}$ is kept. Less value is retained the lower its value. The update gate $z_{t}$ controls the influence of the previous moment’s hidden state $h_{t-1}$ and the current candidate state ${\tilde{h}}_{t}$ on the current hidden state; the more $h_{t-1}$ is maintained, the less ${\tilde{h}}_{t}$ is retained. The formula for each memory cell of the LSTM model can be written as Eq. (A.2) to (A.5):

**Update gate**:

(A.2) $$\begin{array}{c} z_{t}=\phi \left(W_{z}\cdot \left[h_{t-1},x_{t}\right]\right) \end{array}$$

**Reset gate**:

(A.3) $$\begin{array}{c} r_{t}=\phi \left(W_{r}\cdot \left[h_{t-1},x_{t}\right]\right) \end{array}$$

(A.4) $$\begin{array}{c} \tilde{h_{t}}=\tanh\left(W\cdot \left[r_{t}⊙h_{t-1},x_{t}\right]\right) \end{array}$$

(A.5) $$\begin{array}{c} h_{t}=\left(1-z_{t}\right)⊙h_{t-1}+z_{t}⊙\tilde{h_{t}} \end{array}$$

where $h_{t}$ is the hidden state at time $t;x_{t}$ is the GRU input; $\tilde{h_{t}}$ is the candidate state; $W_{z},W_{r}$ and W are the weight matrices to be trained; ϕ is the activation function; and $z_{t}$ and $r_{t}$ represent the update gate and the reset gate, respectively.

### A3 GRUN network and correction module

GRUN is a classic neural network used for single-step water demand forecasting and can realize multi-step output through iterative forecasting. The network structure of GRUN, mainly consists of two parts: the GRU layer and the dense layer. The three GRU layers respectively accept historical water demand from the recent, near, and distant, and model its time dependency. Through the dense layer, the information at different time scales is aggregated to get the prediction for the next time step.

However, due to the inherent error accumulation effect of iterative forecasting, an additional correction module has been proposed to correct the accumulated forecast error when making multi-step forecasts. First, the model generates multi-step forecast results for the future at once through iterative forecasting on the training dataset. Then, a fully connected neural network without an activation function is built to learn a correction weight matrix. The trained correction weight matrix is used to correct the out-of-sample forecast results.

### A4 Kmeans feature expansion

When forecasting water demand, to compensate for the singularity of the input features, the historical water demand data is classified unsupervised based on the Kmeans clustering algorithm. Then, this classification information will be used as a new feature, representing the relative position of the current water demand in the data distribution. Its specific expression can be shown as follows:

(A.6) $$w^{\left(i,j\right)}=\left\{ \begin{array}{cc} 1 & x_{i}\in {\text{Class}}_{j}\\ 0 & \;\text{otherwise}\; \end{array}\right.$$

## References

[bib0001] Adamowski J.F. (2008). Peak daily water demand forecast modeling using artificial neural networks. J. Water Resour. Plan. Manag..

[bib0002] Antunes A., Andrade-Campos A., Sardinha-Lourenço A., Oliveira M. (2018). Short-term water demand forecasting using machine learning techniques. J. Hydroinform..

[bib0003] Bakker M., Vreeburg J., van Schagen K., Rietveld L. (2013). A fully adaptive forecasting model for short-term drinking water demand. Environ. Modell. Softw..

[bib0004] Bergstra J., Bardenet R., Bengio Y., Kégl B. (2011). Algorithms for hyper-parameter optimization. Adv. Neural Inf. Process. Syst..

[bib0005] Caiado, J., 2007. Forecasting water consumption in spain using univariate time series models.

[bib0006] Caiado J. (2010). Performance of combined double seasonal univariate time series models for forecasting water demand. J. Hydrologic Eng..

[bib0007] Candelieri A. (2017). Clustering and support vector regression for water demand forecasting and anomaly detection. Water.

[bib0008] Candelieri A., Giordani I., Archetti F., Barkalov K., Meyerov I., Polovinkin A., Sysoyev A., Zolotykh N. (2019). Tuning hyperparameters of a svm-based water demand forecasting system through parallel global optimization. Comput. Oper. Res..

[bib0009] Chen G., Long T., Xiong J., Bai Y. (2017). Multiple random forests modelling for urban water consumption forecasting. Water Resour. Manag..

[bib0010] Chen L., Yan H., Yan J., Wang J., Tao T., Xin K., Li S., Pu Z., Qiu J. (2022). Short-term water demand forecast based on automatic feature extraction by one-dimensional convolution. J. Hydrol..

[bib0011] Chung J., Gulcehre C., Cho K., Bengio Y. (2014). Empirical evaluation of gated recurrent neural networks on sequence modeling. arXiv preprint arXiv:1412.3555.

[bib0012] Dauphin Y.N., Fan A., Auli M., Grangier D. (2017). International Conference on Machine Learning.

[bib0013] Du B., Zhou Q., Guo J., Guo S., Wang L. (2021). Deep learning with long short-term memory neural networks combining wavelet transform and principal component analysis for daily urban water demand forecasting. Expert Syst. Appl..

[bib0014] Gato S., Jayasuriya N., Hadgraft R., Roberts P. (2005). A simple time series approach to modelling urban water demand. Australasian J. Water Resour..

[bib0015] Gato S., Jayasuriya N., Roberts P. (2007). Temperature and rainfall thresholds for base use urban water demand modelling. J. Hydrol..

[bib0016] Ghiassi M., Zimbra D.K., Saidane H. (2008). Urban water demand forecasting with a dynamic artificial neural network model. J. Water Resour. Plan. Manag..

[bib0017] González-Vidal A., Cuenca-Jara J., Skarmeta A.F. (2019). 2019 IEEE 5th World Forum on Internet of Things (WF-IoT).

[bib0018] Grinsztajn L., Oyallon E., Varoquaux G. (2022). Why do tree-based models still outperform deep learning on typical tabular data?. Adv. Neural Inf. Process. Syst..

[bib0019] Guo G., Liu S., Wu Y., Li J., Zhou R., Zhu X. (2018). Short-term water demand forecast based on deep learning method. J. Water Resour. Plan. Manag..

[bib0020] Haque M.M., Rahman A., Goonetilleke A., Hagare D., Kibria G. (2015). Impact of climate change on urban water demand in future decades: an australian case study. Adv. Environ. Res., Volume 43.

[bib0021] Hochreiter S., Schmidhuber J. (1997). Long short-term memory. Neural Computat..

[bib0022] Hu P., Tong J., Wang J., Yang Y., de Oliveira Turci L. (2019). 2019 IEEE Congress on evolutionary computation (CEC).

[bib0023] Jain A., Kumar Varshney A., Chandra Joshi U. (2001). Short-term water demand forecast modelling at iit kanpur using artificial neural networks. Water Resour. Manag..

[bib0024] Jain A., Ormsbee L.E. (2002). Short-term water demand forecast modeling techniquesconventional methods versus ai. J.-Am. Water Works Assoc..

[bib0025] Ji G., Wang J., Ge Y., Liu H. (2014). The 26th Chinese Control and Decision Conference (2014 CCDC).

[bib0026] Jian C., Gao J., Xu Y. (2022). Anomaly detection and classification in water distribution networks integrated with hourly nodal water demand forecasting models and feature extraction technique. J. Water Resour. Plan. Manag..

[bib0027] Jung D., Kang D., Kang M., Kim B. (2015). Real-time pump scheduling for water transmission systems: case study. KSCE J. Civil Eng..

[bib0028] Kalchbrenner N., Espeholt L., Simonyan K., Oord A.v.d., Graves A., Kavukcuoglu K. (2016). Neural machine translation in linear time. arXiv preprint arXiv:1610.10099.

[bib0029] Kang H.-S., Kim H., Lee J., Lee I., Kwak B.-Y., Im H. (2015). Optimization of pumping schedule based on water demand forecasting using a combined model of autoregressive integrated moving average and exponential smoothing. Water Sci. Technol.: Water Supply.

[bib0030] Kingma D.P., Ba J. (2014). Adam: a method for stochastic optimization. arXiv preprint arXiv:1412.6980.

[bib0031] Kühnert C., Gonuguntla N.M., Krieg H., Nowak D., Thomas J.A. (2021). Application of lstm networks for water demand prediction in optimal pump control. Water.

[bib0032] Luna T., Ribau J., Figueiredo D., Alves R. (2019). Improving energy efficiency in water supply systems with pump scheduling optimization. J. Clean. Prod..

[bib0033] Mouatadid S., Adamowski J. (2017). Using extreme learning machines for short-term urban water demand forecasting. Urban Water J..

[bib0034] Oliveira P.J.A., Boccelli D.L. (2017). World Environmental and Water Resources Congress 2017.

[bib0035] Praskievicz S., Chang H. (2009). Identifying the relationships between urban water consumption and weather variables in seoul, korea. Phys. Geogr..

[bib0036] Pu Z., Yan J., Chen L., Li Z., Tian W., Tao T., Xin K. (2023). A hybrid wavelet-cnn-lstm deep learning model for short-term urban water demand forecasting. Front. Environ. Sci. Eng..

[bib0037] Ribeiro M.T., Singh S., Guestrin C. (2016). Proceedings of the 22nd ACM SIGKDD international conference on knowledge discovery and data mining.

[bib0038] Rinaudo J.-D. (2015). Long-term water demand forecasting. Understand. Manag. Urban Water Trans..

[bib0039] Sainath T.N., Vinyals O., Senior A., Sak H. (2015). 2015 IEEE international conference on acoustics, speech and signal processing (ICASSP).

[bib0040] Salloom T., Kaynak O., He W. (2021). A novel deep neural network architecture for real-time water demand forecasting. J. Hydrol..

[bib0041] Salloom T., Kaynak O., Yu X., He W. (2022). Proportional integral derivative booster for neural networks-based time-series prediction: case of water demand prediction. Eng. Appl. Artific. Intell..

[bib0042] Smolak K., Kasieczka B., Fialkiewicz W., Rohm W., Siła-Nowicka K., Kopańczyk K. (2020). Applying human mobility and water consumption data for short-term water demand forecasting using classical and machine learning models. Urban Water J..

[bib0043] Vijai P., Sivakumar P.B. (2018). Performance comparison of techniques for water demand forecasting. Procedia Comput. Sci..

[bib0044] Xenochristou M., Kapelan Z., Hutton C., Hofman J. (2017). Identifying relationships between weather variables and domestic water consumption using smart metering. Proc. CCWI.

[bib0045] Xu Y., Zhang J., Long Z., Chen Y. (2018). A novel dual-scale deep belief network method for daily urban water demand forecasting. Energies.

